# Factors Predicting the Outcome in Patients With Acute Encephalitis Syndrome: A Retrospective Analysis of Surveillance Data in Bangladesh

**DOI:** 10.1155/nri/2045508

**Published:** 2026-09-29

**Authors:** Dewan Imtiaz Rahman, Faruq Abdulla, Wasik Rahman Aquib, Md Mustafizur Rahman, Muhammad Rashedul Alam, Shadman Sakib Choudhury, Mohammad Rezaul Karim, Arifa Nazneen, Kamal Ibne Amin Chowdhury, Tonmoy Sarkar, Anika Farzin, Fateha Akther Ema, Kajal Chandra Banik, Ayesha Siddika, Mohammad Enayet Hossain, Ahmed Nawsher Alam, Sharmin Sultana, Trevor Shoemaker, Christina Spiropoulou, Mohammed Ziaur Rahman, Sayera Banu, Joel M. Montgomery, Tahmina Shirin, Syed Moinuddin Satter

**Affiliations:** ^1^ icddr,b Dhaka Bangladesh icddrb.org; ^2^ Institute of Epidemiology Disease Control and Research (IEDCR) Dhaka Bangladesh iedcr.org; ^3^ Viral Special Pathogens Branch Centers for Disease Control and Prevention (CDC) Atlanta Georgia USA cdc.gov

**Keywords:** acute encephalitis syndrome (AES), encephalitis, mortality, outcome, predictor

## Abstract

**Introduction:**

Acute encephalitis syndrome (AES) is a severe inflammatory condition affecting the central nervous system, frequently leading to significant morbidity and mortality. Despite advancements in diagnostic techniques, the etiology of AES remains unidentified in a substantial number of cases, particularly in low‐ and middle‐income countries like Bangladesh. Understanding factors that predict outcomes in AES patients is crucial for improving management strategies and patient outcomes.

**Methods:**

We used data from the Nipah surveillance in Bangladesh, analyzing patients admitted with AES to 11 surveillance hospitals across diverse geographical regions in Bangladesh from January 2021 to December 2023. Trained surveillance team members screened patients and collected data through face‐to‐face interviews using a structured questionnaire, and surveillance physicians made decisions on enrollment as an AES case. Bivariate and multivariable logistic regression analyses were used to identify predictors of mortality.

**Results:**

A total of 12,603 AES patients were included, of whom 2,759 (22%) died. Mortality rates varied significantly across different administrative divisions, with the highest rates observed in the Rangpur division. Increased age, female sex, and delayed hospital admission were associated with higher odds of mortality. Patients from divisions like Rangpur and those aged 60 or over had notably higher odds of mortality, with adjusted odds ratios of 2.55 and 4.22, respectively. Clinical factors such as high pulse rate, ronchi, difficulty breathing, altered mental status, stiff neck, paralysis, and unconsciousness at admission were significantly associated with unfavorable outcomes.

**Discussion:**

The findings highlight significant geographical, demographic, and clinical predictors of mortality among AES patients in Bangladesh. Older age, delayed hospital admission, and certain reported clinical symptoms on admission increase the risk of death. The study underscores the importance of timely intervention and targeted management strategies, particularly in identified aforementioned high‐risk groups.

**Conclusion:**

Identifying predictors of adverse outcomes in AES patients can aid in developing early warning and triage systems, potentially enabling adequate resource allocation and the delivery of timely, personalized, and higher standard of care.

## 1. Introduction

Acute encephalitis syndrome (AES), a severe inflammatory condition affecting the central nervous system, is a global concern [1]. In clinical terms, AES is characterized by a prolonged change in mental state persisting for at least 24 h, coupled with indications of inflammation in the brain parenchyma, stemming from infectious, postinfectious, or noninfectious origins [1–4]. According to the World Health Organization (WHO), a clinical case of AES is defined as “a person of any age, at any time of year, presenting with acute onset of fever and at least one of the following: a change in mental status (including symptoms such as confusion, disorientation, coma, or inability to talk) or new onset of seizures (excluding simple febrile seizures)” [5].

As reported by various studies, the estimated annual incidence of encephalitis, encompassing all types, falls within the range of 1–14 cases per 100,000 globally [6–9]. However, despite diagnostic progress, the underlying cause of AES remains undetermined in as many as 85% of cases worldwide [10]. AES imposes considerable mortality, morbidity, and healthcare costs, particularly affecting low‐ and middle‐income countries [11–15].

Treatment options for encephalitis patients are often limited and rely mostly on supportive care. This is due to the uncertainty of etiology, as well as diverse clinical and epidemiological features [16, 17]. Often, guidelines for encephalitis management are focused on the empirical use of broad‐spectrum antimicrobial and anti‐inflammatory therapies, alongside supportive care [18, 19]. However, in countries with a higher prevalence of AES and inadequate diagnostic capabilities, this reliance on empirical treatment can lead to unfavorable patient outcomes [14, 20].

Current knowledge regarding factors influencing severe AES patient outcomes, including death, is limited. Studies conducted outside Bangladesh have shown some associations between certain clinical presentations and increased rates of mortality [21–23]; however, these studies have largely been conducted in high‐income settings with relatively small sample sizes, and few have examined how geographic, demographic, and clinical factors jointly predict AES mortality. In Bangladesh, a country with a substantial AES burden and limited diagnostic capacity, predictors of AES‐related death remain largely unexplored, and no large‐scale, multisite study has previously addressed this gap.

This study addresses this gap by leveraging a large, multisite AES surveillance dataset drawn from 11 hospitals across diverse geographic divisions of Bangladesh over 3 years, representing, evaluating predictors of AES mortality in this setting. By simultaneously examining geographic, demographic, and clinical predictors, we provide the first comprehensive characterization of factors driving AES outcomes in Bangladesh. The findings have the potential to strengthen the early identification of high‐risk AES patients by informing the development of early warning and triage systems at healthcare facilities. The findings also provide a foundation for developing a predictive model of AES prognosis by integrating demographic, epidemiological, and clinical predictors of mortality to aid clinical decision‐making.

## 2. Materials and Methods

### 2.1. Study Setting

We conducted a retrospective analysis of data from all patients admitted with acute encephalitis at 11 surveillance hospitals within the Nipah surveillance in Bangladesh between January 2021 and December 2023. These surveillance hospitals are secondary/tertiary level hospitals (district hospitals are usually termed secondary level hospitals, and the medical college hospitals are termed tertiary level hospitals, covering a diverse geographical location (Figure 1).

**FIGURE 1:**
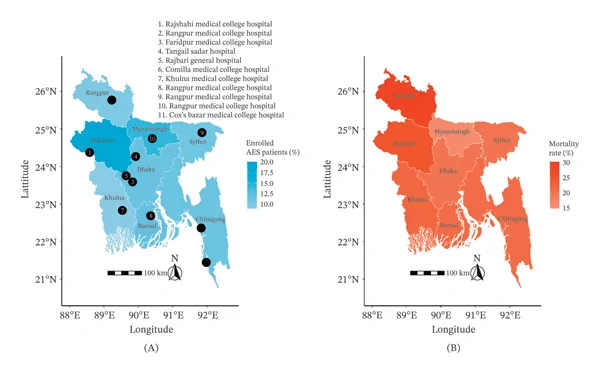
(A) Distribution of enrolled AES patients and location of the surveillance hospitals under the surveillance platform; (B) distribution of mortality rate among the AES patients.

### 2.2. Study Population

#### 2.2.1. AES Inclusion Criteria

As described in the Nipah surveillance [24], patients of any age and sex admitted to the medicine, neuromedicine, and pediatrics departments of surveillance hospitals were considered AES cases if they met the following two criteria:

a. Reported or measured acute fever (axillary temperature > 38.5°C) AND
b. Evidence of acute brain pathology (e.g., altered mental status, new onset seizures, or new neurological deficit either diffuse or localized to the brain).

This definition differs from the WHO AES case definition by specifying a measurable fever threshold (axillary > 38.5°C) and adding new neurological deficits. These changes were adopted to improve specificity by ensuring standardized case identification, capturing atypical neurological presentations, and excluding mild fevers not related to central nervous system involvement. Additionally, during screening, patients diagnosed with simple febrile seizures and those with diagnostic or laboratory evidence of conditions other than acute encephalitis were excluded.

### 2.3. Data

This study used data collected from AES patients enrolled in the national Nipah virus surveillance on the day of admission. As part of the surveillance, trained surveillance team members conducted preliminary screening of patients admitted to the medicine, pediatrics, and neurology departments. Subsequently, surveillance physicians from the respective departments reviewed case profiles, examined patients as needed, and made decisions on enrollment as an AES case. Later, surveillance staff collected demographic information, clinical profiles on admission, and data on some epidemiological indicators, e.g., date of hospitalization and lag time from symptom onset to hospital admission, through face‐to‐face interviews with patients and their attendants using a structured questionnaire.

#### 2.3.1. Response Variable

In this study, patient outcome was treated as the response variable, categorized as “death” or “survival,” based on a combination of in‐hospital mortality and survival status up to 15 days after hospital discharge. In‐hospital deaths were determined from hospital records, while postdischarge survival status was assessed through follow‐up phone calls made 15 days after discharge as part of surveillance activity.

#### 2.3.2. Predictor Variables

This study considered all possible predictor variables available in the dataset, including the demographic characteristics, clinical signs and symptoms, exposure factors, and healthcare received by the patients. A complete list of the considered predictor variables with their categories is summarized in Table 1.

**TABLE 1:** Bivariate analysis of the patients’ outcome with the considered predictor variables.

Patients’ characteristics	Categories	Patients’ outcome		*p*‐value
		Survival *n* (%)	Death *n* (%)	
*Demographic Status*				
Division	Barisal	1186 (78.4)	326 (21.6)	< 0.001
	Chittagong	1145 (78.8)	309 (21.3)	
	Dhaka	1176 (80.0)	295 (20.1)	
	Khulna	920 (77.8)	262 (22.2)	
	Mymensingh	1547 (83.5)	306 (16.5)	
	Rajshahi	1849 (75.3)	606 (24.7)	
	Rangpur	897 (70.6)	373 (29.4)	
	Sylhet	1124 (79.9)	282 (20.1)	

Age (Years)	0–4	3548 (89.8)	405 (10.3)	< 0.001
	5–17	2033 (87.1)	301 (12.9)	
	18–39	1555 (74.8)	525 (25.2)	
	40–59	1253 (67.7)	599 (32.3)	
	> 59	1455 (61.0)	929 (39.0)	

Sex	Male	5787 (79.5)	1497 (20.6)	< 0.001
	Female	4057 (76.3)	1262 (23.7)	

Months of Hospital Admission	January	873 (78.0)	247 (22.1)	0.121
	February	762 (77.5)	221 (22.5)	
	March	784 (76.0)	247 (24.0)	
	April	519 (78.5)	142 (21.5)	
	May	633 (77.2)	187 (22.8)	
	June	788 (80.4)	192 (19.6)	
	July	769 (78.2)	214 (21.8)	
	August	896 (78.6)	244 (21.4)	
	September	898 (79.9)	226 (20.1)	
	October	1058 (79.7)	270 (20.3)	
	November	989 (78.0)	279 (22.0)	
	December	875 (75.1)	290 (24.9)	

*Physiological Parameters*				
Pulse/Heart Rate	Normal: 60–90	3381 (71.3)	1359 (28.7)	< 0.001
	Low: < 60	25 (78.1)	7 (21.9)	
	High: > 90	6438 (82.2)	1393 (17.8)	

Respiratory Rate	Normal	4504 (74.8)	1515 (25.2)	< 0.001
	Low	239 (82.1)	52 (17.9)	
	High	5101 (81.1)	1192 (18.9)	

*Observed Symptoms*				
Ronchi	Present	90 (59.2)	62 (40.8)	< 0.001
	Absent	9754 (78.3)	2697 (21.7)	

Rales	Present	91 (68.4)	42 (31.6)	0.007
	Absent	9753 (78.2)	2717 (21.8)	

Wheezing	Present	144 (60.5)	94 (39.5)	< 0.001
	Absent	9700 (78.5)	2665 (21.6)	

Fever	Present	9844 (100)	2759 (100)	—

Muscle Pain	Present	386 (74.1)	135 (25.9)	0.023
	Absent	9458 (78.3)	2624 (21.7)	

Joint Pain	Present	170 (73.3)	62 (26.7)	0.072
	Absent	9674 (78.2)	2697 (21.8)	

Rash	Present	57 (74.0)	20 (26.0)	0.385
	Absent	9787 (78.1)	2739 (21.9)	

Vomiting	Present	4232 (79.1)	1117 (20.9)	0.019
	Absent	5612 (77.4)	1642 (22.6)	
Diarrhea	Present	591 (83.5)	117 (16.5)	< 0.001
	Absent	9253 (77.8)	2642 (22.2)	

Cough	Present	1421 (80.9)	335 (19.1)	0.002
	Absent	8423 (77.7)	2424 (22.4)	

Sore Throat	Present	140 (78.7)	38 (21.4)	0.860
	Absent	9704 (78.1)	2721 (21.9)	

Difficulty of Breathing	Present	1184 (65.1)	636 (35.0)	< 0.001
	Absent	8660 (80.3)	2123 (19.7)	

Disorientation^∗^	Present	6806 (74.7)	2311 (25.4)	< 0.001
	Absent	3038 (87.2)	448 (12.9)	

Altered Mental Status^∗^	Present	6116 (73.8)	2168 (26.2)	< 0.001
	Absent	3728 (86.3)	591 (13.7)	

Stiff Neck	Present	3430 (73.7)	1224 (26.3)	< 0.001
	Absent	6414 (80.7)	1535 (19.3)	

Convulsion	Present	7570 (81.5)	1722 (18.5)	< 0.001
	Absent	2274 (68.7)	1037 (31.3)	

Paralysis	Present	41 (45.6)	49 (54.4)	< 0.001
	Absent	9803 (78.3)	2710 (21.7)	

Unconscious	Present	5855 (72.1)	2265 (27.9)	< 0.001
	Absent	3989 (89.0)	494 (11.0)	

Salivation	Present	3052 (79.9)	767 (20.1)	0.001
	Absent	6792 (77.3)	1992 (22.7)	

^∗^Altered mental status was used as a broad clinical term covering any variation from normal consciousness, including confusion, lethargy, agitation, or unresponsiveness, while disorientation specifically referred to patients who were conscious but unable to correctly identify person, place, or time.

### 2.4. Statistical Analysis

Frequency and percentage were determined for the categorical variables distributed by the considered response variable. The chi‐squared test was used to examine the bivariate association between the categorical predictor variables and the response variable. The normality of symptom onset‐to‐admission lag time was evaluated using the Anderson–Darling test (significance threshold set at *p* < 0.05). As the data showed a non‐normal distribution, medians and interquartile ranges (IQRs) were calculated, and differences between survivors and nonsurvivors were compared using the Mann–Whitney *U* test. The predictor variables with a *p*‐value of less than 0.2 in the bivariate association with the response variable were considered for the next step of multivariable logistic regression modeling. A significance threshold of *p* < 0.20 at the bivariate stage was used to reduce the risk of prematurely excluding potentially important predictors, an approach recommended in logistic regression model‐building [25]. In addition, this less common cutoff point was used to capture broader trends that might have been missed [26, 27]. Before performing multivariable logistic regression modeling, multicollinearity among the considered predictor variables was assessed using generalized variance inflation factor (GVIF) adjusted by corresponding degrees of freedom (DF). A stepwise selection procedure (combining forward and backward selection) based on the Akaike Information Criterion (AIC) was used to identify the most parsimonious set of predictor variables for the multivariable logistic regression model, an approach commonly applied in epidemiological studies with a large number of candidate predictors [25]. Multicollinearity was assessed among candidate predictors prior to model building, and model robustness was evaluated using 10‐fold cross‐validation to help address known limitations of stepwise methods [28]. Finally, a multivariable binary logistic regression model was performed to predict the unfavorable patient outcome called death with the considered predictor variables and to determine the corresponding odds ratio (OR) along with a 95% confidence interval (CI). In the multivariable binary logistic regression modeling, the cutoff point of the *p*‐value of 0.05 was considered at a 5% level of significance. The performance of the final model was assessed using overall classification accuracy and area under the receiver operating characteristic (ROC) curve. In addition, model calibration was assessed using the calibration intercept (calibration‐in‐the‐large), calibration slope, and Brier score. A 10‐fold cross‐validation was used to ensure the model’s robustness and to provide a reliable estimate of its predictive performance across different subsets of the data. All the analyses of this study were performed in STAT v15 and R v 4.3.2.

This manuscript was prepared in accordance with the STROBE (Strengthening the Reporting of Observational Studies in Epidemiology) guidelines for cross‐sectional studies [29]. A completed STROBE checklist is provided in Table 2.

**TABLE 2:** STROBE‐checklist‐v4‐combined‐plosmedicine: short details.

	Item no.	Recommendation	Page no.
Title and abstract	1	(a) Indicate the study’s design with a commonly used term in the title or the abstract	1–3
		(b) Provide in the abstract an informative and balanced summary of what was done and what was found	1–3

*Introduction*			
Background/rationale	2	Explain the scientific background and rationale for the investigation being reported	4‐5
Objectives	3	State specific objectives, including any prespecified hypotheses	5

*Methods*			
Study design	4	Present key elements of study design early in the paper	5
Setting	5	Describe the setting, locations, and relevant dates, including periods of recruitment, exposure, follow‐up, and data collection	5
Participants	6	(a) Cohort study—Give the eligibility criteria, and the sources and methods of selection of participants. Describe methods of follow‐up	6
		Case‐control study—Give the eligibility criteria, and the sources and methods of case ascertainment and control selection. Give the rationale for the choice of cases and controls	
		Cross‐sectional study—Give the eligibility criteria, and the sources and methods of selection of participants	
		(b) Cohort study—For matched studies, give matching criteria and number of exposed and unexposed	N/A
		Case–control study—For matched studies, give matching criteria and the number of controls per case	
Variables	7	Clearly define all outcomes, exposures, predictors, potential confounders, and effect modifiers. Give diagnostic criteria, if applicable	7
Data sources/measurement	8^∗^	For each variable of interest, give sources of data and details of methods of assessment (measurement). Describe comparability of assessment methods if there is more than one group	6‐7
Bias	9	Describe any efforts to address potential sources of bias	N/A
Study size	10	Explain how the study size was arrived at	N/A
Quantitative variables	11	Explain how quantitative variables were handled in the analyses. If applicable, describe which groupings were chosen and why	7
Statistical methods	12	(a) Describe all statistical methods, including those used to control for confounding	7‐8
		(b) Describe any methods used to examine subgroups and interactions	7‐8
		(c) Explain how missing data were addressed	7–8
		(d) Cohort study—If applicable, explain how loss to follow‐up was addressed	7‐8
		Case–control study—If applicable, explain how matching of cases and controls was addressed	
		Cross‐sectional study—If applicable, describe analytical methods taking account of sampling strategy	
		(e) Describe any sensitivity analyses	7‐8

*Results*			
Participants	13^∗^	(a) Report numbers of individuals at each stage of study—e.g., numbers potentially eligible, examined for eligibility, confirmed eligible, included in the study, completing follow‐up, and analyzed	9–11
		(b) Give reasons for nonparticipation at each stage	N/A
		(c) Consider use of a flow diagram	N/A
Descriptive data	14^∗^	(a) Give characteristics of study participants (e.g., demographic, clinical, social) and information on exposures and potential confounders	9–11
		(b) Indicate number of participants with missing data for each variable of interest	N/A
		(c) Cohort study—Summarize follow‐up time (e.g., average and total amount)	N/A
Outcome data	15^∗^	Cohort study—Report numbers of outcome events or summary measures over time	N/A
		Case–control study—Report numbers in each exposure category, or summary measures of exposure	N/A
		Cross‐sectional study—Report numbers of outcome events or summary measures	9
Main results	16	(a) Give unadjusted estimates and, if applicable, confounder‐adjusted estimates and their precision (e.g., 95% confidence interval). Make clear which confounders were adjusted for and why they were included	9–11
		(b) Report category boundaries when continuous variables were categorized	N/A
		(c) If relevant, consider translating estimates of relative risk into absolute risk for a meaningful time period	N/A
Other analyses	17	Report other analyses done—e.g., analyses of subgroups and interactions, and sensitivity analyses	N/A

*Discussion*			
Key results	18	Summarize key results with reference to study objectives	12–15
Limitations	19	Discuss limitations of the study, taking into account sources of potential bias or imprecision. Discuss both direction and magnitude of any potential bias	15–16
Interpretation	20	Give a cautious overall interpretation of results considering objectives, limitations, multiplicity of analyses, results from similar studies, and other relevant evidence	12–15
Generalizability	21	Discuss the generalizability (external validity) of the study results	12–15

*Other information*			
Funding	22	Give the source of funding and the role of the funders for the present study and, if applicable, for the original study on which the present article is based	17

*Note:* STROBE Statement—checklist of items that should be included in reports of observational studies. An Explanation and Elaboration article discusses each checklist item and gives methodological background and published examples of transparent reporting. The STROBE checklist is best used in conjunction with this article (freely available on the websites of PLoS Medicine at https://www.plosmedicine.org/, Annals of Internal Medicine at https://www.annals.org/, and Epidemiology at https://www.epidem.com/). Information on the STROBE Initiative is available at https://www.strobe-statement.org.

^∗^Give information separately for cases and controls in case–control studies and, if applicable, for exposed and unexposed groups in cohort and cross‐sectional studies.

### 2.5. Ethics

The research protocol of the Nipah surveillance was reviewed and approved by the ethical committee of icddr,b. Informed consent was obtained from all suspected acute encephalitis cases enrolled in the surveillance or their next of kin.

## 3. Results

Initially, data for 12,637 individuals were retrieved from the surveillance database. After excluding records with missing data or implausible values, data from 12,603 individuals were considered for the final analysis. The proportion of encephalitis was slightly higher among males (57.8% vs 42.2%). In terms of age distribution, almost one‐third of patients were under 5 years old. The demographic characteristics of the encephalitis patients are shown in Figure 2.

**FIGURE 2:**
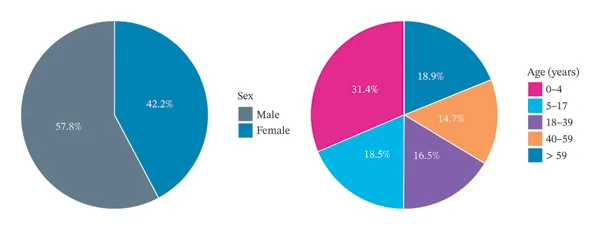
Demographic characteristics of the patients.

Overall mortality, encompassing deaths during hospitalization and within 15 days postdischarge, was used as the primary outcome measure. Approximately 22% (2759/12,603) of patients enrolled with suspected AES died. The outcome of encephalitis patients varied significantly (*p*‐value: < 0.001) across different administrative divisions at a 5% level of significance. Rangpur division exhibited the highest death rate (about 29%). In other divisions, an approximately 20%–22% death rate was observed. Furthermore, age and sex had a significant association with the patients’ outcome (*p*‐value: < 0.001). An increased mortality rate was observed with the increasing age of the patients. High mortality was found among the patients aged over 59 years (around 39%), while those aged 0–4 years had the lowest death rate of about 10%. Females had a slightly higher mortality rate (23.7%) compared to their counterparts (20.6%). There was no significant difference in mortality across the months of hospital admission (Table 1).

Pulse and respiratory rates were found to be significantly associated with patient outcome (*p*‐value: < 0.001). All the observed symptoms except rash and sore throat showed statistically significant association with patient outcome. Patients who developed ronchi, wheezing, difficulty in breathing, disorientation, altered mental status, paralysis, or unconsciousness exhibited nearly twice the mortality rate compared to those who did not exhibit these symptoms (Table 1). The lag time from symptom onset to hospital admission did not follow a normal distribution (*p*‐value: < 0.001). The median lag time (from symptom onset to hospital admission) was 2 days for both survivors (IQR: 1–4) and nonsurvivors (IQR: 1–5). Although the medians were identical, the distribution of lag times differed significantly between the survivors and nonsurvivors (*p* < 0.001); patients who died had a substantially wider spread and longer lag times than survivors.

We found no potential multicollinearity among the considered predictor variables, as the adjusted GVIF values ranged from 1.01 to 1.29. The next step of the analysis was to fit the binary logistic regression model, with the patient outcome as the dependent variable and the patient characteristics identified from the bivariate analysis as independent variables. Potential predictors were selected using a stepwise selection procedure, achieving a minimum AIC value of 11,640. The selected variables are listed in Table 3. The overall classification accuracy of the final model was 78.5%, which remained consistent after 10‐fold cross‐validation with the overall mean classification accuracy across cross‐validation folds of 78.4% (range: 77.7%–79.8%). The area under the curve for the final model was 0.75, and the Brier score was 0.149. In addition, model calibration showed no systematic over‐ or underestimation of the predicted probabilities with a calibration intercept of approximately 0 (3.66 × 10^−12^) and exhibited excellent agreement between predicted and observed risks with a calibration slope of 1.

**TABLE 3:** Results of multivariable logistic regression.

Characteristics	Levels	Adjusted odds ratio (95% CI)	*p*‐value
Division	Barisal	Reference	
	Chittagong	1.78 (1.46, 2.17)	< 0.001
	Dhaka	1.38 (1.14, 1.68)	0.001
	Khulna	1.41 (1.15, 1.73)	< 0.001
	Mymensingh	1.53 (1.27, 1.86)	< 0.001
	Rajshahi	1.53 (1.29, 1.81)	< 0.001
	Rangpur	2.55 (2.10, 3.09)	< 0.001
	Sylhet	1.60 (1.31, 1.95)	< 0.001

Age (Years)	0–4	Reference	
	5–17	1.02 (0.86, 1.21)	0.825
	18–39	2.15 (1.82, 2.56)	< 0.001
	40–59	3.04 (2.55, 3.62)	< 0.001
	> 59	4.22 (3.54, 5.02)	< 0.001

Sex	Male	Reference	
	Female	1.14 (1.04, 1.25)	0.005

Lag Time from Symptom Onset to Hospital Admission (Days)		1.04 (1.03, 1.05)	< 0.001

Pulse Rate	Normal	Reference	
	Low	0.92 (0.34, 2.19)	0.850
	High	1.18 (1.06, 1.31)	0.003

Ronchi	Absent	Reference	
	Present	1.64 (1.13, 2.37)	0.008

Diarrhea	Absent	Reference	
	Present	0.84 (0.67, 1.04)	0.119

Difficulty of Breathing	Absent	Reference	
	Present	2.08 (1.84, 2.35)	< 0.001

Altered Mental Status	Absent	Reference	
	Present	1.16 (1.03, 1.31)	0.014

Stiff Neck	Absent	Reference	
	Present	1.18 (1.07, 1.29)	< 0.001

Convulsion	Absent	Reference	
	Present	0.81 (0.72, 0.90)	< 0.001

Paralysis	Absent	Reference	
	Present	2.93 (1.87, 4.61)	< 0.001

Unconscious	Absent	Reference	
	Present	2.66 (2.37, 2.98)	< 0.001

The adjusted ORs, along with their corresponding 95% CIs and significance values, are presented in Table 3. At the 5% level of significance, patients from all divisions exhibited significantly higher odds of mortality compared to the Barisal division. Rangpur division exhibited 2.55 times higher odds of mortality compared to Barisal. As age increases, the odds of mortality due to encephalitis significantly increase. Patients aged 40–59 years had approximately 3 times higher odds of mortality compared to those aged 0–4 years, while patients over 59 years old had the highest odds of more than 4‐fold. The odds of mortality in the age group of 5–17 years were not statistically different from the age group of 0–4 years. Female patients had a slightly increased (14%) odds of mortality compared to their counterparts. Furthermore, for each additional day of delay in hospital admission following symptom onset, the odds of mortality increased by 4% (Table 3).

The findings indicated that around 18% higher odds of mortality was observed among the patients who had a high pulse rate compared to those with a normal pulse rate. Furthermore, patients with paralysis and unconsciousness showed about 3 times and 2.7 times higher odds of mortality, respectively. In addition, patients who had difficulty breathing were twice as likely to die. Moreover, patients with, ronchi, altered mental status, and stiff neck had significantly higher odds of mortality by 64%, 16%, and 18%, respectively, compared to those patients who did not have these symptoms. However, those who had convulsions had 19% lower odds of mortality (OR: 0.81, 95% CI: 0.72–0.90, *p* < 0.001) compared to their counterparts. However, the presence of diarrhea showed no significant association with increased rates of mortality (Table 3). A detailed comparison of the ORs is provided in Figure 3.

**FIGURE 3:**
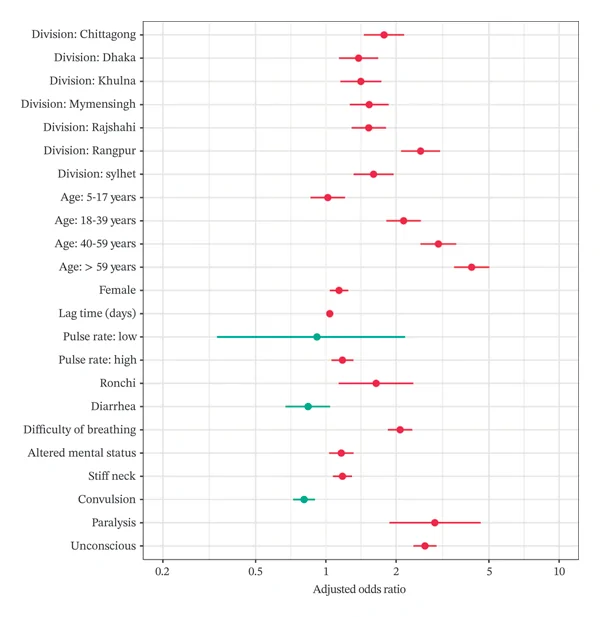
Comparison of adjusted ORs with 95% CI.

## 4. Discussion

In our enrollment of 12,603 patients with AES, 2759 patients died. Geographical location, age, and sex were among the numerous negative mortality predictors identified in this investigation. Furthermore, the poor prognosis of AES patients was associated with factors such as a longer delay from symptom onset to hospital admission, high pulse rate, ronchi, difficulty breathing, altered mental status, stiff neck, paralysis, and unconsciousness at admission. The association with a stiff neck is of particular neurological significance, as it is a classic clinical sign of meningeal irritation, indicating that the inflammatory process has extended to involve the meninges [30]. Consistent with previous evidence, patients with meningoencephalitis typically experience worse clinical outcomes and higher mortality than those with encephalitis alone, owing to more extensive central nervous system involvement and complications [31, 32]. However, patients with convulsions and diarrhea had some lower odds of mortality. This may be due to misclassification under the broad AES case definition, which could have included patients with nonencephalitic diarrhea and convulsions. As a result, some cases presenting with convulsions and diarrhea may have had a less severe disease course than those with true encephalitis, contributing to the lower observed mortality in these subgroups.

The unfavorable outcomes of AES patients admitted to surveillance hospitals significantly differ across administrative regions (divisions) in Bangladesh. Specifically, AES patients from the Rangpur division had the highest chance of death. AES patients from Dhaka and Khulna divisions had similar chances of death, as did AES patients from Mymensingh and Rajshahi divisions. This variation may be attributed to differences in the causes and etiologies of AES across different geographic locations in Bangladesh. A previous study reported that the causes of encephalitis, meningoencephalitis, and meningitis can vary substantially across the geographic location [33, 34]. This regional disparity is likely multifactorial, with etiological heterogeneity being a primary consideration. The known causes of encephalitis, such as Japanese encephalitis and Nipah virus, exhibit distinct geographic and ecological niches. However, these pathogens account for only a small fraction of enrolled AES cases in national surveillance [24, 35], indicating that the majority of etiologies remain unidentified. It is possible that patients in the Rangpur division were affected by severe or treatment‐resistant pathogens.

In addition, variations in the standard of care across different surveillance hospitals may impact the unfavorable outcomes of AES patients who are admitted. We were unable to identify any published studies systematically describing the quality or availability of AES‐related clinical management across these hospitals. The Bangladesh Health Facility Survey 2022 reported hospital readiness indicators for maternal and child health and noncommunicable diseases, but did not include any domains related to acute infectious or neurological illnesses other than tuberculosis [36]. In our analysis, patients admitted to hospitals in the Dhaka division had statistically lower odds of unfavorable outcomes compared to patients from other divisions. This observation may be due to the fact that surveillance hospitals in the Dhaka division are located near the capital, where there are more tertiary‐level government and private health facilities [36, 37], potentially leading to more referrals of severe cases before enrollment into the surveillance. Additionally, it is plausible that patients with severe disease were more likely to be admitted to these tertiary hospitals, which could introduce referral bias. Variations in healthcare‐seeking behaviors, socioeconomic status, and access to emergency care within the division might also confound the relationship between the division of admission and AES outcomes.

The chance of unfavorable outcomes increases with the age of AES patients admitted to surveillance hospitals. Older individuals often have more comorbidities, which can complicate their condition and increase mortality risk. Aging leads to a decline in immune function, making older patients more susceptible to infections like encephalitis [38, 39]. In addition, early diagnosis of encephalitis can be challenging, especially in older adults, because symptoms may overlap with the age‐related conditions; they are more likely to have altered consciousness, confusion, and impaired cognitive function [38]. Age was identified as a risk factor that was independently associated with a poor prognosis for encephalitis patients during their hospitalization in a previous study [40]. The study conducted at Mayo Clinic Rochester (United States of America) by Singh also documented the same result using multivariate regression. Results indicated that those 65 years of age or older have poor prognosis in cases of acute encephalitis [41].

Female AES patients admitted to surveillance hospitals are significantly more susceptible to poor outcomes. The reason for this finding may be that females in Bangladesh are less likely to seek health care than males [42, 43], which could lead to delayed care‐seeking and worse outcomes.

The prolonged lag time from symptom onset to hospital admission in encephalitis patients is a significant predictor of poor outcomes due to accelerated disease progression, delayed proper treatment management, exacerbated immune responses, increased risk of secondary complications, extensive brain damage, and continued neurological deterioration. A previous study reported that patients with autoimmune encephalitis who delayed hospitalization after their first symptom onset were more critically ill compared to those who sought hospitalization sooner [44]. Moreover, a study conducted in Bangladesh reported that hospital admissions are commonly delayed due to cost, underestimation of illness severity by family members, and the absence of someone to accompany the patient [45]. This emphasizes the critical importance of timely hospital admission in reducing encephalitis‐related mortality.

Several clinical symptoms recorded on admission, such as high pulse rate, ronchi, difficulty breathing, altered mental status, stiff neck, paralysis, and unconsciousness, increase the odds of an unfavorable outcome for AES patients. Electrocardiographic abnormalities, such as increased heart rate, are commonly observed in viral infections [46]. A study reported that approximately 90% of patients presenting with shock upon admission died, whereas only 19% of those without shock on admission expired [47]. Moreover, Glasgow Coma Scale (GCS) score (neurological scale used to assess the level of consciousness) of < 13 and seizure significantly increase the risk of adverse clinical outcomes of patients with encephalitis [38]. In our study, among all enrolled cases, 31% were children under the age of 4 years. Febrile seizures or convulsions are relatively common in this age group, affecting approximately 2–4% of children at least once before the age of 5 years [48, 49]. Our analysis revealed that convulsions were associated with 19% lower odds of mortality. Several explanations may account for this finding. First, the observed connection between convulsions and lower mortality might be due to potential lead‐time bias. As described by previous studies, convulsions could trigger quick medical attention and lead to earlier recognition by caregivers and transfer to healthcare facilities [50, 51]. Second, there is no established evidence linking sudden unexpected death in epilepsy (SUDEP) with febrile seizures [52], suggesting that the mortality risk typically associated with seizures in other clinical contexts may not directly apply to this AES cohort. Taken together, these factors may explain the apparent protective association of convulsions with mortality observed in our study cohort; however, we acknowledge that residual confounding by symptom severity or underlying etiology cannot be fully excluded.

From a clinical perspective, these findings have important implications for the initial assessment and management of patients presenting with AES. Because the etiological diagnosis of AES is frequently unavailable at the time of admission in many resource‐limited settings, readily identifiable demographic characteristics, delay in hospital presentation, and routine clinical signs can help clinicians recognize patients with higher chance of death. Patients presenting with older age, female sex, prolonged symptom‐to‐admission interval, altered mental status, unconsciousness, paralysis, respiratory distress, stiff neck, or tachycardia may benefit from closer neurological monitoring, earlier supportive management, timely referral to higher‐level facilities when indicated, and prioritization of limited critical care resources. At the public health level, our findings also highlight the need to improve community awareness of early symptoms and strengthen referral pathways to reduce delays in hospital admission, which may ultimately improve survival among patients with AES. Finally, as the underlying cause of AES remains unidentified in the majority of cases, our findings support the value of a syndromic and sign‐based early warning approach, which may be particularly useful in low‐resource settings where advanced diagnostics are not readily available.

### 4.1. Limitations

This study has a few limitations. Firstly, our study includes secondary‐ and tertiary‐level hospitals; therefore, the patients enrolled might have had more severe conditions and worse outcomes. This introduces a potential referral bias and potentially underrepresents patients with milder illnesses who mainly seek care at primary healthcare facilities. Secondly, we used the broad case definition of AES for case identification because of the unavailability of pathogen‐specific confirmation due to resource constraints and the lack of advanced diagnostic tests in many participating hospitals. This led to case enrollment mainly based on clinical suspicion of encephalitis, and we cannot rule out nonencephalitic causes of fever with altered mental status or seizures among included participants. Also, the case definition covers all‐cause encephalitis, including infectious (bacterial, viral, or parasitic) and noninfectious (autoimmune) cases, and limitations in identifying etiologic agents decreased the accuracy of case characterization and the interpretation of results. Thirdly, some potentially important variables, such as comorbidities, were not available in the dataset. Their absence may have introduced incomplete adjustment for confounding, which limits the interpretability of the observed associations between predictor variables and mortality.

## 5. Conclusion

Our study identified several demographic factors and clinical characteristics as early predictors of mortality among patients with AES in Bangladesh. This study provided the opportunity to establish early warning scores or systems at primary healthcare facilities to identify AES patients who require advanced care at referral hospitals. Future research should aim to generate evidence that deepens the understanding of long‐term AES outcomes and sequelae, in relation to etiological diagnoses, the evolution and timing of clinical features throughout the disease course, comorbidities, and complications developed during the acute episode, the need for mechanical ventilation, as well as the treatment and care received, which will create scope to develop a robust predictive model of the prognostic factors of AES patients. This model could provide a framework to aid clinical decision‐making by identifying AES patients likely to be at a higher risk of poor outcomes through an efficient triage system in hospitals, enabling adequate resource allocation and the delivery of timely, personalized, and higher standard of care.

## Author Contributions

Dewan Imtiaz Rahman and Faruq Abdulla contributed equally to this work and shared first authorship (joint first author); Dewan Imtiaz Rahman, Faruq Abdulla: conceptualization, methodology, statistical analysis, visualization, interpretation, writing–original draft, and writing–review and editing; Wasik Rahman Aquib and Md Mustafizur Rahman: data curation, statistical analysis, interpretation, and writing–review and editing; Muhammad Rashedul Alam and Shadman Sakib Choudhury: data curation, interpretation, and writing–review and editing; Mohammad Rezaul Karim, Arifa Nazneen, Kamal Ibne Amin Chowdhury, Tonmoy Sarkar, Anika Farzin, Fateha Akther Ema, Kajal Chandra Banik, Ayesha Siddika, Mohammad Enayet Hossain, Ahmed Nawsher Alam, Sharmin Sultana, Mohammed Ziaur Rahman, Sayera Banu, and Tahmina Shirin: writing–review and editing; Trevor Shoemaker, Christina Spiropoulou, and Joel M. Montgomery: funding acquisition and critical revision of the manuscript; Syed Moinuddin Satter: conceptualization, supervision, writing–review and editing, and critical revision of the manuscript.

## Funding

This study has been conducted as a part of a cooperative agreement between the US Centers for Disease Control and Prevention and the icddr,b, Bangladesh.

## Disclosure

All authors read and approved the final version of the manuscript. The findings and conclusions in this report are those of the author(s) and do not necessarily represent the official position of [the Centers for Disease Control and Prevention]. The authors report no relevant disclosures.

## Conflicts of Interest

The authors declare no conflicts of interest.

## Data Availability

The data supporting the findings of this study are obtained from an ongoing Nipah surveillance in Bangladesh. During consent process, participants were assured that their personal identifiers such as patient names, admission dates, locations, and GPS coordinates would remain confidential and would not be made publicly available. According to icddr,b data protection policies and ethical obligations, the full dataset cannot be shared in the manuscript, supplemental files, or through a public repository, as it would risk disclosure of confidential information and unpublished data from ongoing studies. However, nonidentifiable data specific to this manuscript may be made available to qualified researchers upon reasonable request.
